# The prevalence, trends, and geographical distribution of human papillomavirus infection in China: The pooled analysis of 1.7 million women

**DOI:** 10.1002/cam4.2017

**Published:** 2019-07-27

**Authors:** Bo Zhu, Yunyong Liu, Tingting Zuo, Xiaoli Cui, Mengdan Li, Jing Zhang, Huihui Yu, Haozhe Piao

**Affiliations:** ^1^ Department of Cancer Prevention and Treatment Cancer Hospital of China Medical University/Liaoning Cancer Hospital & Institute Shenyang People's Republic of China; ^2^ Department of Gynecologic Oncology Cancer Hospital of China Medical University/Liaoning Cancer Hospital & Institute Shenyang People's Republic of China

**Keywords:** China, distribution, general population, HPV

## Abstract

Human papillomavirus (HPV) infection which continues to be the most common sexually transmitted disease, has been identified as a major risk factor for cervical cancer. Therefore, it is very important to understand and grasp the distribution of HPV in Chinese population, and make the foundation for the development of cervical cancer vaccine in China. An extensive search strategy was conducted in multiple literature databases. All retrieved studies were screened by October 31, 2018. The prevalence of HPV infection was analyzed using random effects model. A total of 68 studies satisfied the inclusion criteria for our study. The national overall prevalence of HPV infection was 15.54% (95% CI: 13.83%‐17.24%). we also performed subgroup analysis by age, geographic location, level of economic development, HPV assay method, and type of HPV infection. The top 5 common HPV types detected in general population, were HPV 16 (3.52%, 95% CI: 3.18%‐3.86%), 52 (2.20%, 95% CI: 1.93%‐2.46%), 58 (2.10%, 95% CI: 1.88%‐2.32%), 18 (1.20%, 95% CI: 1.05%‐1.35%), and 33 (1.02%, 95% CI: 0.89%‐1.14%). Except for the higher prevalence of HPV infection in 2009 and 2010, the prevalence of HPV infection in other years changed little, ranged from 13.2% to 17.4%. HPV type in Chinese women was quite distinctive. HPV infection played a critical role in the occurrence of cervical cancer, understanding the distribution of HPV type and performing the HPV type testing had important clinical value for colposcopy referral and increasing the detection rate. Therefore, our findings could provide evidence for cervical cancer screening and vaccine, in order to reduce the burden of cervical cancer.

## 1 INTRODUCTION

Cervical cancer is one of the most common malignancies among women worldwide. According to Global Cancer Statistics in 2012, an estimated 527 600 new cases and 265 700 deaths were found in women worldwide; cervical cancer is recognized as the fourth most common cancer and the fourth leading cause of cancer‐related death in women and have been a major public health problem.^1^The burden of cervical cancer in developing countries is much higher than that in developed countries. The incidence of cervical cancer was not the top 10 in developed countries, but the second in the developing countries. The mortality was the ninth in the developed countries, and the third in developing countries.^1^ Based on data in 2011‐2015 from USA, the number of new cases was 7.4 per 100 000 women per year, and the number of deaths was 2.3 per 100 000 women per year. In terms of the latest National Cancer Statistics in China, the number of new cases was 10.08 per 100 000 women per year, and the number of deaths was 2.98 per 100 000 women per year.^2^

Human papillomavirus (HPV) infection continues to be the most common sexually transmitted disease, has been identified as a major risk factor for cervical cancer. Eighty per cent of people will get an HPV infection in their life. HPV infection continues to cause significant burden on women. Most HPV infections are asymptomatic, transient, and will clear within 1‐2 years, but those that persist can progress to precancer or cancer.^3^ Many epidemiologic studies have found that nearly 100% of patients with cervical cancer have HPV infection.^4^, ^5^, ^6^ Sexually transmitted HPV types is usually identified as two categories: (a) High‐risk HPV, which mainly include HPV 16, 18, 31, 33, 35, 39, 45, 51, 52, 56, 58, 59, 66, and 68, are strongly associated with cervical cancer. Two of these, HPV 16 and 18, are responsible for most HPV‐caused cancers. (b) Low‐risk HPV, which mainly include HPV 6, 11, 30, 42, 43, 44, and 61, and HPV 6 and 11 cause 90% of all genital warts. HPV testing is more sensitive than cytology in the screening of cervical cancer.

As the largest developing country in the world, China has about 130 000 new cases of cervical cancer each year, accounting for about 1/4 of new cases in the world. If effective measures are not taken, it is estimated that the number of new cases of cervical cancer will increase to 187 000 by 2050.^7^ China has actively promoted the screening and prevention of cervical cancer in recent years, but the incidence and mortality of cervical cancer have remained high, and the age distribution becomes younger. Currently, the vaccines for cervical cancer are mainly targeted at tetravalent vaccine (HPV 16, 18, 6, and 11) and bivalent vaccine (HPV 16 and 18), based on the data from HPV epidemiological survey in Europe or the United States.^8^ It needs to be considered whether the vaccines are suitable for Chinese population. It is very important to understand and grasp the distribution of HPV in Chinese general population, which provides scientific basis for prevention and treatment of cervical cancer. In 2018, Zhou HL and Xu HH et al respectively published a systematic review on prevalence and distribution of HPV in patients, which has invasive carcinoma of cervix (ICC), high‐grade squamous intraepithelial lesion (HSIL), or low‐grade squamous intraepithelial lesion (LSIL).^8^, ^9^ These studies mainly studied the prevalence and distribution of HPV in patients, but did not study on general population; the results might be influenced by selection bias, which cannot indeed reflect the real situation of HPV infection in general population. Therefore, we perform this study to integrate the findings of 68 population‐based studies. The aim was to explore that (a) temporal trends and geographical patterns in the HPV infection epidemic and (b) the differences between china and foreign countries.

## 2 METHODS

### 2.1 Search strategy

An extensive search strategy was conducted in multiple literature databases by October 31, 2018. The literature databases included PubMed, Web of Science, Chinese Scientific Journals Full text Database (CQVIP), China National Knowledge Infrastructure (CNKI), and Wanfang Data. We set the following key words: (a) “Papillomaviridae” and free word “China” were used for PubMed and Web of Science; (b) “Papillomaviridae” was set as subject heading (including title, abstract, and keyword) to search Chinese database. We also reviewed reference lists from all the relevant original research and review studies to identify additional possibly eligible studies. This study was conducted and reported in accordance with the Preferred Reporting Items for Systematic Reviews and Meta‐Analyses (PRISMA) statement issued in 2009.^10^

### 2.2 Study selection

We used Endnote^®^ (version X6; Thomson Reuters, Inc, Philadelphia, PA) bibliographic software to create an electronic library, which collected all the retrieved studies from literature databases. After deleting the duplicated studies by the software identification, 2 independent authors (Bo Zhu, Tingting Zuo) performed title/abstract screening and full text screening in turn. When any disagreements happened, 3 independent authors (Bo Zhu, Tingting Zuo, and Mengdan Li) reached agreement by discussion.

The inclusion and exclusion criteria were as follows: (a) the subjects of studies should come from the general population, excluded the studies that contained outpatients and/or patients; (b) for the detection of HPV infection, both high‐risk and low‐risk types must be included; (c) the methodology of HPV detection should be explicitly stated; (d) the study contained the prevalence of HPV infection, or provided the available data to calculate the estimates; (e) when 2 studies were found to be from the same population, the latest or maximum sample size article was included.

### 2.3 Quality assessment

The methodological quality and validity of the included studies were assessed by an existing tool, which was modified to assess risk of bias in prevalence studies.^11^ Ten aspects in the tool were used to evaluate the quality of the included study, which was shown in supplementary Data S1. The included studies were assessed a score of 0 (high risk) or 1 (low risk) for each aspect. The score of study quality ranged from 0‐10. The study with 8‐10 points was regarded as “high quality”, the study with 4‐7 points was regarded as “moderate quality”, and otherwise, the study was regarded as “low quality”. The quality assessment tool is shown in supplementary Data S1.

### 2.4 Data extraction

The included studies were independently read in detail by two reviewers (Bo Zhu and Tingting Zuo). Two reviewers used a fixed table to extract the information, which included the following: first author and year of publication, sample size, age, study period, region classification, and province.

### 2.5 Statistical analysis

Stata software package (Version 12.0; Stata Corp., College Station, TX) was used in the pooled analysis. The random effects model (DerSimonian‐Laird) was used to pool the study‐specific estimates to obtain an overall summary estimate of the prevalence of HPV infection. In order to fully reflect the distribution of HPV infection in China, we performed subgroup analysis by age, geographic location, the level of economic development, HPV assay method, and type of HPV infection. In geographic location, studies were categorized into 1 of 6 geographical locations according to the traditional Chinese administrative regions: East China, Northeast, North China, South China, Central China, Northwest, and Southwest. In economic development level, studies were categorized as 3 groups by per capita GDP in 2016, which included low, moderate, and high level. HPV assay method mainly contained PCR & hybridization, real‐time PCR, and sequencing PCR. Type of HPV infection included high risk HPV or low risk HPV, single infection or multiple infection, and the top 10 HPV infection subtypes.

## 3 RESULTS

### 3.1 Search results

**Figure 1:**
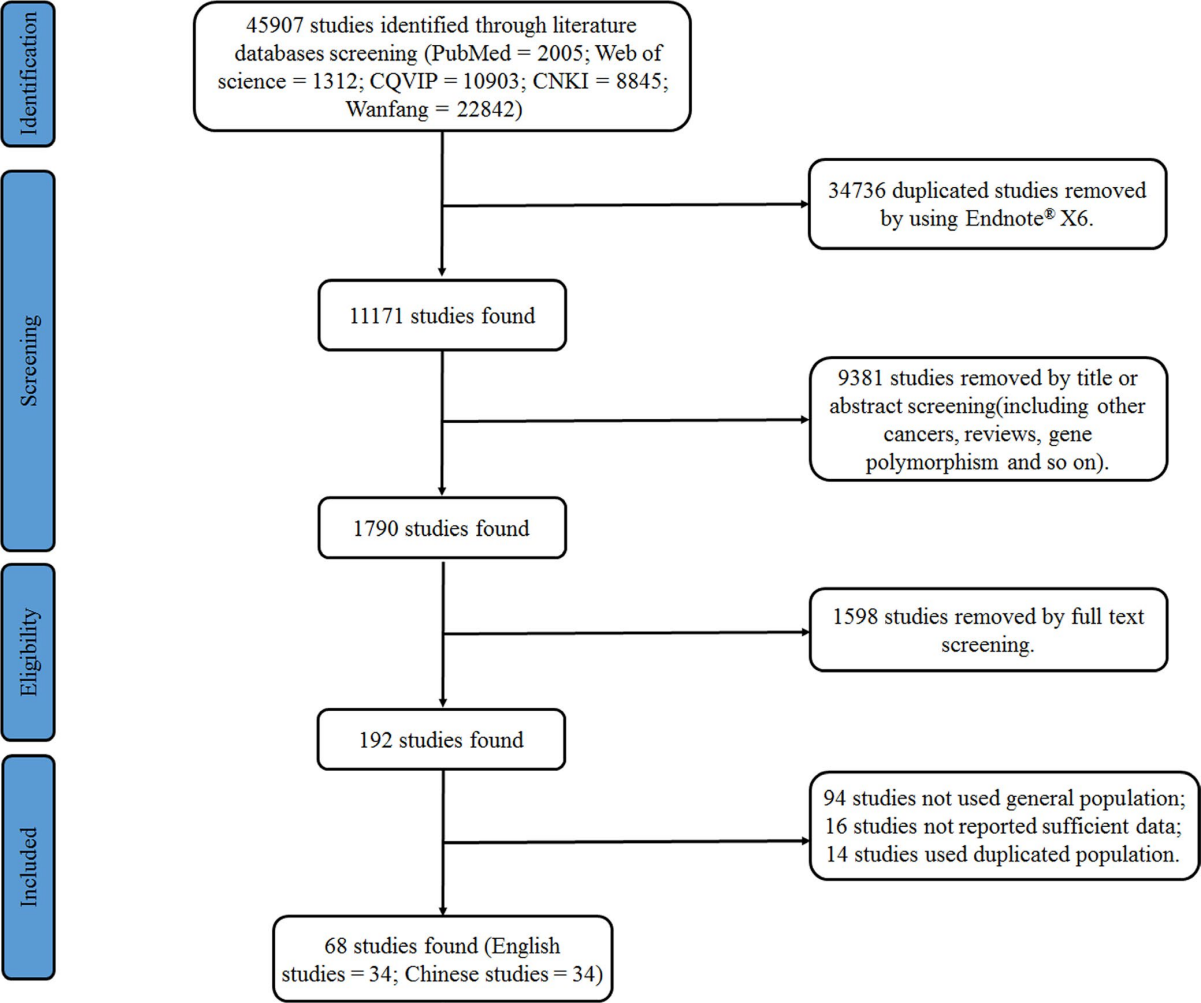
The flow chart of screening process in our meta‐analysis

A total of 45 907 studies were found through searching the database (MEDLINE: 2005, Web of Science: 1312, CQVIP: 10903, CNKI: 8845 and Wanfang: 22842). First, the Endnote® bibliographic software was used to identify the duplicated studies, 34 736 studies were removed. Second, 9381 studies were removed by reading title or abstracts, which included the studies related to HPV and cancers, reviews, and genetic polymorphisms. Third, 1598 studies were removed by reading full text. In the remaining 192 papers, a total of 94 studies contained outpatients and/or patients, 16 studies did not provide sufficient data to calculate the prevalence of HPV, and 14 studies were from the same population. Finally, the remaining 68 population‐based studies (English studies: 34; Chinese studies: 34) satisfied the inclusion criteria for our study. A flow chart of the screening process is shown in Figure 1.

### 3.2 The characteristics of the included studies

**Figure 2:**
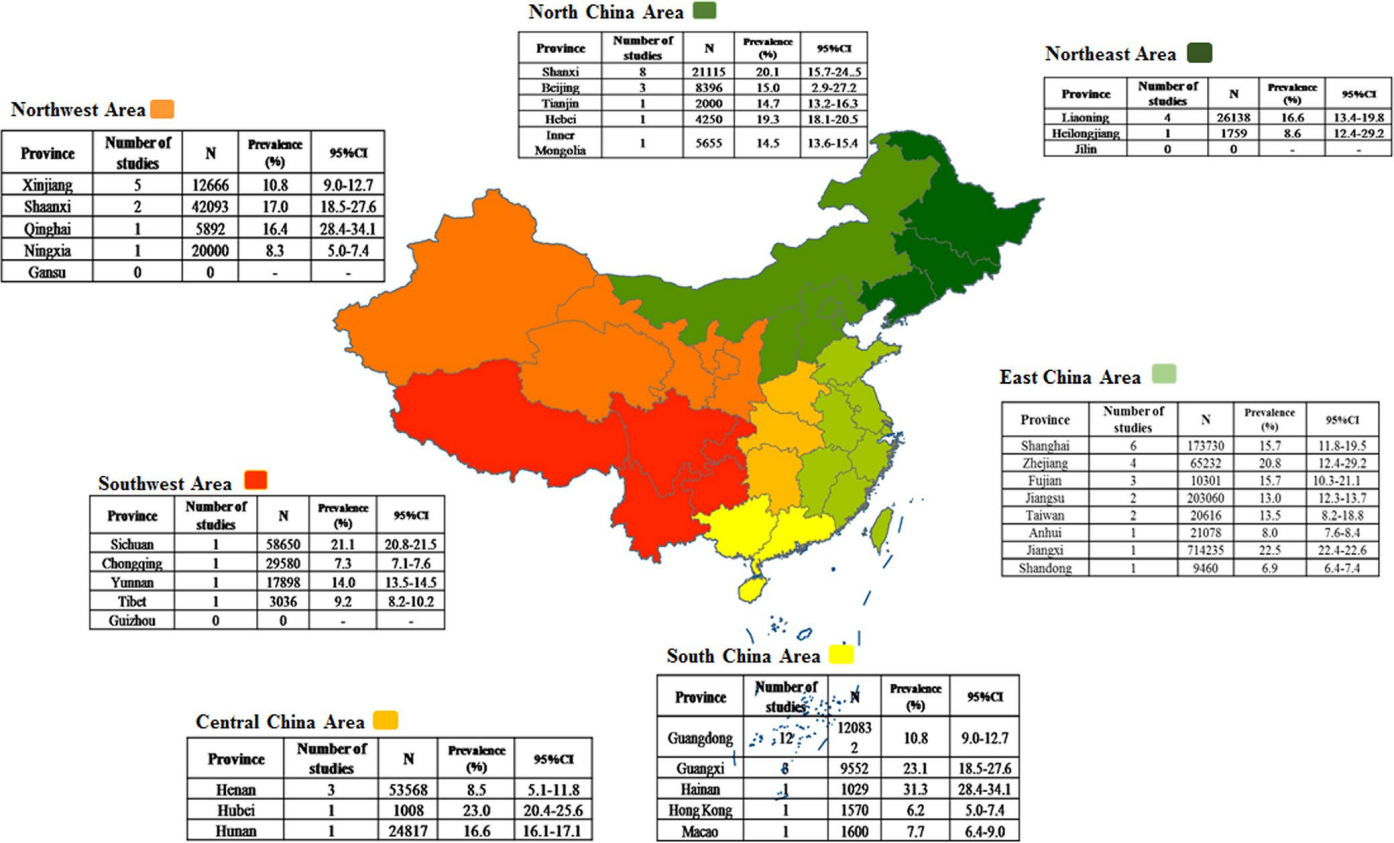
The number of studies, the sample size, and the prevalence on Human papillomavirus (HPV) infection in each province of China

In the included studies, we found there were 31 provinces which reported the prevalence of HPV infection in general population, except for Gansu, Jilin, and Guizhou. The number of studies reported in 10 provinces was over 3. A total of 1705956 people were included in our study, the number of people in each province ranged from 1008 to 714 235. The prevalence of HPV infection ranged from 6.2% (Hong Kong) to 31.3% (Hainan). The characteristic of the included studies was in Table 1. Figure 2 showed the number of studies, the sample size, and the prevalence on HPV infection in each province.

**Table 1:** The characteristic of the included studies

Author	Year of publication	Number of subjects	Age	Study period	The definition of HPV POSITIVITY	The specimen type	HPV assay method	Region classification	Province	City	The quality score
Jin, LM	2018	116441	21‐78	2013‐2016	HPV DNA	Exfoliated cells	Sequencing PCR	city	Shanghai		**9**
Zhang, C	2018	33562	11‐96	2016.1‐2016.12	HPV DNA	Exfoliated cells	PCR & hybridization	city	Shanghai		9
Wang, X	2018	5655	17‐68	2014.4‐2015.12	HPV DNA	Exfoliated cells	PCR & hybridization	city	Inner Mongolia		9
Dong, L	2017	1997	35‐45	1999.6	HPV DNA	Exfoliated cells	PCR & hybridization	rural	Shanxi	Xianghua	9
Zhou, X. H	2017	2452	20‐80	2013.3‐2013.12	HPV DNA	Exfoliated cells	PCR & hybridization	city	Shanghai		9
Xu, H. H	2017	37967	15‐90	2012.12‐2016.2	HPV DNA	Exfoliated cells	Real‐time PCR	city	Zhejiang	Taizhou	8
Zhong, TY	2017	714235	16‐77	2010.8‐2015.12	HPV DNA	Exfoliated cells	PCR & hybridization	city	Jiangxi	Nanchang, Jiu‐jiang, Yingtan, Jingdezhen, Shangrao, Yichun, Xinyu, Pingxiang,Fuzhou, Ji'an, and Ganzhou	9
Wu, X	2017	2300	18‐45	2013.3‐2013.7	HPV DNA	Exfoliated cells	PCR & hybridization	city	Guangxi	Liuzhou	9
Abulizi, G.	2017	6000	21‐60		HPV DNA	Exfoliated cells	Real‐time PCR	rural	Xinjiang	Uyghur	9
Zhang, J	2017	58650	13‐81	2012.6‐2016.5	HPV DNA	Exfoliated cells	PCR & hybridization	city	Sichuan	Chengdu	8
Qi, ZW	2017	4250	18‐69	2013.1‐2016.1	HPV DNA	Exfoliated cells	PCR & hybridization	city and rural	Hebei	Chengde	8
Shen, XP	2017	20000	35‐64	2015	HPV DNA	Exfoliated cells	Real‐time PCR	rural	Ningxia		7
Zhang Xiaohong	2017	38408	Not reported	2012.3‐2015.12	HPV DNA	Exfoliated cells	PCR & hybridization	city and rural	Shaanxi		7
Yang, DM	2017	9001	22‐84	2016.1‐2016.12	HPV DNA	Exfoliated cells	PCR & hybridization	city	Hunan	Changsha	8
Jin, DC	2017	50502	18‐50	2015.1‐2015.12	HPV DNA	Exfoliated cells	Real‐time PCR	city	Henan	Zhengzhou	8
Baloch, Z	2016	17898	18‐93	2013.10‐2015.2	HPV DNA	Exfoliated cells	Sequencing PCR	city and rural	Yunnan		9
Gu, Y	2016	10501	39‐55	2014.1‐2015.5	HPV DNA	Exfoliated cells	PCR & hybridization	city	Shanghai		9
Liu, F	2016	2187	25‐65	2007‐2009	HPV DNA	Exfoliated cells	Sequencing PCR	rural	Henan	anyang	9
Wei, F	2016	2378	18‐55	2014.5‐2014.7	HPV DNA	Exfoliated cells	Real‐time PCR	city	Guangxi	Liuzhou	8
Zeng, XX	2016	8284	18‐65	2012.1‐2012.12	HPV DNA	Exfoliated cells	Sequencing PCR	city	Guangdong	Heyuan	9
Niyazi, M	2016	883	15‐54	2006.9	HPV DNA	Exfoliated cells	PCR & hybridization	city	Xinjiang	Hetian	8
Zhang, Y	2016	3000	30‐59	2015.3‐2015.6	HPV DNA	Exfoliated cells	PCR & hybridization	city	Guangdong	Shenzhen	8
Que, M	2016	21078	35‐64	2014	HPV DNA	Exfoliated cells	PCR & hybridization	rural	Anhui		8
Ji,HY	2016	5892	18‐80	2013.10‐2015.10	HPV DNA	Exfoliated cells	PCR & hybridization	city and rural	Qinghai		9
Long, Xin	2016	29580	16‐81	2010.11‐2015.9	HPV DNA	Exfoliated cells	PCR & hybridization	city and rural	Chongqing		9
Wang, JX	2015	191829	35‐65	2013	HPV DNA	Exfoliated cells	PCR & (mass‐spectrometric detection) MSD	city	Jiangsu	Wuxi	8
Mijit, F	2015	4500	20‐69	2008.6‐2008.9	HPV DNA	Exfoliated cells	PCR & hybridization	city	Xinjiang	Hetian	8
Zhao, Y	2015	6273	17‐59	2004.5‐2007.4	HPV DNA	Exfoliated cells	PCR & hybridization	city and rural	—	Beijing, Shanghai, Shenzhen, Shenyang, Shanxi, Henan, Xinjiang	10
Chen, X	2015	2000	21‐65	2013.4‐2013.10	HPV DNA	Exfoliated cells	Sequencing PCR	city	Tianjin		8
Hong, H	2015	1373	22‐64	2012.4‐2012.6	HPV DNA	Exfoliated cells	PCR & hybridization	rural	Zhejiang	Ningbo	8
Yang, L	2015	9,460	Not reported	Not reported	HPV DNA	Exfoliated cells	Not reported	city	Shandong	Weihai	8
Zhao, Q	2015	24817	18‐80	2012.7‐2013.7	HPV DNA	Exfoliated cells	PCR & hybridization	city	Hunan		9
Li, XL	2015	1008	23‐78	2014.8‐2014.10	HPV DNA	Exfoliated cells	PCR & hybridization	city	Hubei	Yichang	9
Chen, MH	2014	11231	25‐74	2012.1‐2012.12	HPV DNA	Exfoliated cells	Sequencing PCR	city	Jiangsu	Wuxi	8
Jing, L	2014	78355	18‐75	2011.5‐2012.11	HPV DNA	Exfoliated cells	Real‐time PCR	city	Guangdong		9
Zhu, MH	2014	7796	18‐70	2012.3‐2012.7	HPV DNA	Exfoliated cells	PCR & hybridization	city	Guangdong	Huizhou	8
Pei, YY	2014	1944	20‐67	Before 2014	HPV DNA	Exfoliated cells	PCR & hybridization	city	Guangdong	Shenzhen	7
Yang, L	2013	1759	24‐77	2010.6‐2010.10	HPV DNA	Exfoliated cells	Real‐time PCR	city	Heilongjiang	Daqing	8
Wu, EQ	2013	4,215	17‐54	2006.5‐2007.4	HPV DNA	Exfoliated cells	PCR & hybridization	city and rural	—	Beijing, Shanghai, Shanxi, Henan, and Xinjiang	10
Zhang, R	2013	10000	17‐89	2011.3‐2011.5	HPV DNA	Exfoliated cells	PCR & hybridization	rural	Shanghai	Fengxian	9
Wu, XQ	2013	4228	19‐59	2011.11‐2012.4	HPV DNA	Exfoliated cells	PCR & hybridization	city	Guangdong	Guangzhou	8
Weng, ZC	2013	1029	Not reported	2010.10‐2012.5	HPV DNA	Exfoliated cells	PCR & hybridization	city	Hainan	Haikou	7
Wu, D	2013	2050	15‐91	2010.1‐2012.11	HPV DNA	Exfoliated cells	PCR & hybridization	city	Beijing		8
Wang, S	2012	24041	18‐60	2007‐2010	HPV DNA	Exfoliated cells	PCR & hybridization	city	Liaoning		9
Wang, Y	2012	3052	20‐79	2011.3‐2012.3	HPV DNA	Exfoliated cells	PCR & hybridization	city	Fujian		8
ZhangJing	2012	3685	29‐67	2010.7‐2011.6	HPV DNA	Exfoliated cells	PCR & hybridization	rural	Shaanxi		8
Chen, JC	2012	3399	26‐59	2010.1‐2011.4	HPV DNA	Exfoliated cells	PCR & hybridization	city	Guangdong	Shantou	8
Wang, YY	2012	9683	Not reported	2006.4‐2010.4	HPV DNA	Exfoliated cells	PCR & hybridization	city	Guangdong	Shenzhen	7
Zhao, R	2011	5552	25‐54	2006.9‐2008.12	HPV DNA	Exfoliated cells	PCR & hybridization	city and rural	Beijing		9
Liu, SS	2011	1570	Not reported	2007.1‐2008.4	HPV DNA	Exfoliated cells	PCR & hybridization	city	Hong Kong		9
Ablimit, T	2011	400	26‐65	2008.7‐2008.9	HPV DNA	Exfoliated cells	PCR & hybridization	rural	Xinjiang	Hetian	7
Liu, JX	2011	4874	24‐55	2008.7‐2010.12	HPV DNA	Exfoliated cells	PCR & hybridization	city and rural	Guangxi		8
Chen HC	2011	10602	30‐65	1991‐1992	HPV DNA	Exfoliated cells	PCR & hybridization	city	Taiwan		9
Yip, Y. C	2010	1600	20‐60	2008.5‐2008.8	HPV DNA	Exfoliated cells	PCR & hybridization	city	Macao		8
Ye, J	2010	4987	20‐79	2007.11‐2008.8	HPV DNA	Exfoliated cells	PCR & hybridization	rural	Zhejiang		9
Wu, D	2010	2338	20‐70	2008.5‐2009.5	HPV DNA	Exfoliated cells	PCR & hybridization	city	Fujian	Fuzhou	9
Jiang, Y	2010	4911	20‐69	2008.4‐2008.9	HPV DNA	Exfoliated cells	PCR & hybridization	city and rural	Fujian	Xiamen	8
Zhouyuan	2010	20905	14‐84	2008.10‐2009.3	HPV DNA	Exfoliated cells	PCR & hybridization	city and rural	Zhejiang		9
Jin, Q	2009	3036	19‐65	2007.8	HPV DNA	Exfoliated cells	PCR & hybridization	city	Xizang	Lhasa, Shigatse, Nagqu	9
Li, N	2008	2374	15‐59	2004.5‐2006.9	HPV DNA	Exfoliated cells	PCR & hybridization	city and rural		Shanxi, Guangdong, Liaoning	10
Chao A	2008	10014	30‐	2004.6‐2005.6	HPV DNA	Exfoliated cells	PCR & hybridization	city	Taiwan		9
Zhang, X	2007	702	19‐59	2006.8‐2006.9	HPV DNA	Exfoliated cells	PCR & hybridization	city	Liaoning	Shenyang	9
Dai, M	2006	662	15‐59	2004.3‐2004.4	HPV DNA	Exfoliated cells	PCR & hybridization	rural	Shanxi		9
Li, LK	2006	685	15‐59	2005.6‐2005.7	HPV DNA	Exfoliated cells	PCR & hybridization	city	Liaoning	Shenyang	9
Shi, JF	2006	1137	15‐59	Before 2006	HPV DNA	Exfoliated cells	PCR & hybridization	city	Guangdong	Shenzhen	9
Wu, KH	2004	500	21‐45	1999.7‐2001.6	HPV DNA	Exfoliated cells	PCR & hybridization	city	Guangdong	Guangzhou	8
Chen Feng	2004	3233	30‐50	2002	HPV DNA	Exfoliated cells	PCR & hybridization	rural	Shanxi		8
Shen Yanhong	2003	9683	30‐50	Before 2003	HPV DNA	Exfoliated cells	PCR & hybridization	rural	Shanxi		8

Note

Because the number of references was too much, the authors made the references of the included studies in Supplementary Data S2.

We assessed the quality of included studies using the modified quality assessment tool. The quality scores of the included studies were shown in Table 1. The quality scores ranged from 7 to 11 and 10. Only 6 studies were assessed as moderate quality, and all other studies were assessed as high quality.

### 3.3 The infection of HPV in Chinese women

In the included studies, there were 71 databases that reported the overall prevalence of HPV infection, the national overall prevalence of HPV infection was 15.54% (95% CI: 13.83%‐17.24%); there were 50 databases that reported HPV infection in urban, the prevalence of HPV infection was 14.86% (95% CI: 12.84%‐16.88%), and there were 21 databases that reported HPV infection in rural, the prevalence of low risk HPV infection was 13.70% (95% CI: 11.18%‐16.22%).

According to the traditional geographical regions of China, we have divided China into seven regions, which included East China, North China, Central China, South China, Southwest, Northwest, and Northeast, the prevalence of HPV infection were 15.99% (95% CI: 12.84%,‐19.13%), 18.43% (95% CI: 13.39%‐23.46%), 12.96% (95% CI: 8.44%‐17.48%), 13.51% (95% CI: 11.34%‐15.68%), 12.92% (95% CI: 5.31%‐20.53%), 12.57% (95% CI: 8.49%‐16.65%), and 19.85% (95% CI: 3.46%‐36.23%), respectively. According to the level of GDP per capita in 2016, China was categorized into 3 groups, namely, low, middle, and high, the prevalence of HPV infection were 16.72%(95% CI: 13.81%‐19.62%), 18.01%(95% CI: 12.67%‐23.35%), and 13.41%(95% CI: 11.74%‐15.09%). According to HPV assay method, the prevalence of HPV infection was 16.55% by PCR and hybridization and the prevalence of HPV infection was 11.73% by sequencing or real‐time PCR.

There were 66 databases that reported the prevalence of high risk HPV infection, the prevalence of high risk HPV infection was 11.93% (95% CI: 10.93%‐12.92%), and there were 46 databases that reported the prevalence of low risk HPV infection, the prevalence of low risk HPV infection was 2.78% (95% CI: 2.35%‐3.20%); there were 35 databases that reported the prevalence of single HPV infection, the prevalence of single HPV infection was 11.00% (95% CI: 9.32%‐12.68%), and there were 48 databases that reported the prevalence of multiple HPV infection, the prevalence of multiple HPV infection was 3.44% (95% CI: 2.69%‐4.18%), the multiple HPV infection included dual infection (2.30%, 95% CI: 2.01%‐2.58%), triple infection (0.51%, 95% CI: 0.42%‐0.60%), fourfold infection (0.11%, 95% CI: 0.07%‐0.15%), and fivefold infection (0.04%, 95% CI: 0.01%‐0.07%), respectively. In the detection of the HPV subtype, we found that HPV16, 52, 58, 18, and 33 are the top 5 subtypes, and the prevalence of the top 5 subtypes was 3.52% (95% CI: 3.18%‐3.86%), 2.20% (95% CI: 1.93%‐2.46%), 2.10% (95% CI: 1.88%‐2.32%), 1.20% (95% CI: 1.05%‐1.35%), 1.02% (95% CI: 0.89%‐1.14%).

We further stratified the analysis by age, found that there were 12 databases which reported the prevalence of high risk HPV infection by age. Because of different age groups in databases, 12 databases were divided into 3 groups for analysis. In group 1, the prevalence of HPV infection in <25, 55‐59, 60‐65 age groups were higher with 13.31%, 14.93%, and 16.33%, respectively, and that in 25‐29, 30‐34 age groups were lower with 9.97% and 9.50%, respectively. In group 2, the prevalence of HPV infection in <25, >65 age groups were higher with 19.21% and 13.33%, respectively, and that in 25‐34, 55‐64 age groups were lower with 9.95% and 9.84%, respectively. In group 3, the prevalence of HPV infection in <25, >60 age groups were higher with 33.37% and 26.03%, respectively, and that in 31‐40, 41‐50 age groups were lower with 13.94% and 13.86%, respectively.

**Figure 3:**
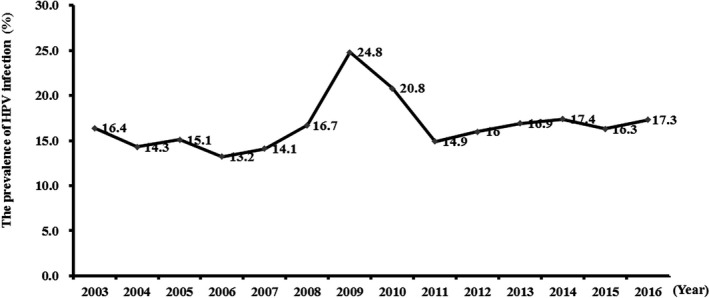
The time trend of Human papillomavirus (HPV) infection between 2003 and 2016

In the included studies, the year of the epidemiological investigation ranged from 1991 to 2016. There was only one study that reported 1991, 1992, 1999, 2000, 2001, 2002, and 2003, the number of studies which reported other years were at least 4 or more. Therefore, we pooled 7 datasets that reported 1991, 1992, 1999, 2000, 2001, 2002, and 2003, to get more stable result. Except for the higher prevalence of HPV infection in 2009 and 2010, the prevalence of HPV infection in other years changed little, ranged from 13.2% to 17.4%. Figure 3 showed the time trend of HPV infection between 2003 and 2016.

## 4 DISCUSSION

The prevalence of HPV infection varied geographically, and the prevalence of cervical infection with human papillomavirus (HPV) in women also varies greatly worldwide. In order to understand the situation of HPV infection in Chinese women accurately, we conducted a pooled analysis of 68 population‐based studies, which contained 1 705 956 subjects. As far as we know, this is the most comprehensive and extensive, HPV‐related study in China. The national overall prevalence of HPV infection was 15.54% (95% CI: 13.83%‐17.24%), which ranged from 6.2% (Hong Kong) to 31.3% (Hainan) and varied by region. This may be due to the large Chinese population and its territories. Meanwhile, age, economic conditions, cultural habits, and population migrations might also influence factors.^12^ Therefore, we further carried out subgroup analysis by different regions and economic levels. The results from our study indicated that the prevalence of HPV infection in urban was similar to that in rural. A high burden of HPV infection was found in East, North, and Northeast region of China. The higher prevalence of HPV infection was found in middle and high economic level. The prevalence of high risk HPV infection, the prevalence of high risk HPV infection (11.93%) was as 4 times as high as that of low risk HPV infection (2.78%). We also analyzed the HPV infection epidemiological trend between 2003 and 2016, and found that the prevalence of HPV infection were higher in 2009 and 2010, the prevalence of HPV infection in other years changed little, ranged from 13.2% to 17.4%.

The prevalence of single infection was 11.00% (95% CI: 9.32‐%‐12.68%) while the prevalence of multiple infection was 3.44% (95% CI: 2.69%‐4.18%). In the multiple HPV infection individuals, the frequencies of 5, 4, 3, and 2 genotypes were 0.04%, 0.11%, 0.51%, and 2.30%, respectively. We found that the main form of HPV infection was single infection, and multiple HPV infections were less common, the results were consistent with previous studies. Some studies had suggested that multiple HPV infections could potentially have competitive and/or cooperative interactions between HPV genotypes. Multiple HPV infections would increase the duration of infection, and patients with multiple high viral loads showed a fourfold to sixfold increased risk of cervical precancerous cytological lesions compared with patients with single high viral loads. There was an association between infection with multiple HPV types and an increased risk of cervical cancer. The mechanisms and potential oncogenic effects of multiple infections warrant further investigation, which could be useful in the development of HPV prophylactic vaccines.

In 2009, on the evidence of epidemiological and biological research, International Cancer Research Institute (IARC) defined 12 HPV types as HPV 16, 18, 31, 33, 35,39, 45, 51, 52, 58, and 59 as carcinogenic HPV (I carcinogen), and defined HPV 68 as a potentially carcinogenic HPV (2A carcinogen). HPV 26, 53, 66, 67, 67, 82, etc, which may be carcinogenic, were defined as Carcinogenic HPV (2B carcinogen).The carcinogenic HPV clustered in 4 species groups of the alpha genus, which included HPV 51 in species 5, HPV 56 in species 6, HPV 18, 39, 45, and 59 in species 7 and HPV 16, 31, 33, 35, 52, and 58 in species 9.^13^ Based on WHO worldwide data, the 5 most frequent HPV types in the general female population are HPV 16, 18, 31, 58, and 52. HPV 16, 18, 58, 31, and 33 are the most common in America, while the 5 most frequent HPV types in Europe were HPV 16, 18, 31, 33, and 58.^14^ In our study, HPV 16, 52, 58, 18, and 33 are the top 5 HPV infection subtypes in China, the distribution of which was different from other regions in the world. We also found that except HPV 18, HPV 16, 52, 58, and 33 was in species 9 of the alpha genus. HPV 16 and HPV 18 are well known as oncogenic genotypes and account for about 70% of invasive cervical cancers (ICC) worldwide. Though more variation in the ranking of individual types was in different countries, HPV 33, 45, 31, 58, 52, and 35 make up the rest of the top 8.^15^ For the Chinese women, Bao YP, et al found that in high‐grade squamous intraepithelial lesions (HSIL) and low‐grade squamous intraepithelial lesions (LSIL) samples, HPV 16, 58, 52, 18, and 33 were the most commonly detected types after adjusting for confounding factors (geographic area, classification of cervical disease status, and HPV testing method).^16^Zhou HL, et al found that in invasive carcinoma of cervix (ICC), the top 5 common HPV types were HPV 16, 18, 58, 52, and 33 in descending order of frequency. The present prophylactic vaccine for cervical cancer is developed based on the results of the HPV epidemiological survey abroad. In China, the quadrivalent vaccine (HPV 16, 18, 6, and 11) and the bivalent vaccine (HPV 16 and 18) were licensed. In our study, the top 5 common HPV types were HPV 16, 18, 58, 52, and 33 in Chinese general population. Therefore, the quadrivalent and bivalent vaccines were unable to cover the common HPV infection types in China, could not provide effective protection for cervical cancer.

In age group, we found that the prevalence of HPV infection in <20 and <25 age groups were high, then there was a distinct decline. When the age reached 60 or even older, the prevalence of HPV infection had increased again. The high prevalence of HPV infection in younger women may be related to unhealthy sexual habits such as premature sexual life, excessive frequency, and excessive sexual partners.^17^ Although the prevalence of HPV infection in young women is high, most of HPV infection may be cleared automatically within 1‐2 years, so the prevalence of HPV infection would be reduced. The immune ability declined with age in old women, especially in the premenopausal and postmenopausal women, the ability in eliminating previous and new infections weakened, so the high prevalence of HPV infection was also in older women.^18^

There were several limitations in our meta‐analysis. First, because our study aims to reflect the HPV prevalence in general population. In the process of detecting HPV for cervical cancer screening, cervical high‐grade lesions, and cancer might be screened out, we should analyze the prevalence of HPV in cervical high‐grade lesions and cancer. But, in process of collecting the included studies, we found that only a few studies reported the number of cervical high‐grade lesions and cancer, fewer studies reported the prevalence of HPV in cervical high‐grade lesions and cancer was too small, most of studies did not report the prevalence of HPV infection in cervical high‐grade lesions and cancer. We could not analyze the prevalence of HPV infection in cervical high‐grade lesions and cancer. Second, different detection methods might have different effects on the detection rate of HPV infection, and might affect the prevalence of HPV infection. Therefore, we performed subgroup analysis by the HPV assay method, the prevalence of HPV infection was 16.55% by PCR & hybridization, and the prevalence of HPV infection was 11.73% by sequencing or real‐time PCR. The HPV assay method might be the heterogeneity source of the prevalence of HPV infection, it deserved our attention in the follow‐up studies. Third, in our study, we had considered the effects of many factors on the prevalence of HPV infection in our study. Because our included studies did not report standardized rate, when we merged the prevalence of HPV infection, we just only focused on the description of the results, could not compare.

In conclusion, HPV type in Chinese women was quite distinctive. HPV infection played a critical role in the occurrence of cervical cancer, which affected by geographical region, economic conditions, cultural habits, and population migrations. In the screening process of cervical cancer, we should pay attention to mental stress, destruction of the cervical tissue by invasive operation and economic burden caused by over diagnosis and treatment, when improving the sensitivity of screening and reducing the missed diagnosis rate were emphasized. Understanding the distribution of HPV type and performing the HPV type testing had important clinical value for colposcopy referral and increasing the detection rate. Therefore, our findings could provide evidence for cervical cancer screening and vaccine, in order to reduce the burden of cervical cancer.

## CONFLICT OF INTEREST

None declared.

5

**Table 2:** The pooled prevalence of Human papillomavirus (HPV) infection in Chinese women

	Number of databases	Number of Subjects	Prevalence (%)	95% CI
Overall	71	1705956	15.54	13.83‐17.24
Subgroup Analysis				
Areas				
Urban	50	1488733	14.86	12.84‐16.88
Rural	21	113946	13.70	11.18‐16.22
East China	18	1197096	15.99	12.84‐19.13
North China	13	35761	18.43	13.39‐23.46
Central China	5	79393	12.96	8.44‐17.48
South China	20	155199	13.51	11.34‐15.68
Southwest	4	109164	12.92	5.31‐20.53
Northwest	9	80651	12.57	8.49‐16.65
Northeast	6	33552	19.85	3.46‐36.23
GDP				
High	36	631416	13.41	11.74‐15.09
Middle	15	178795	18.01	12.67‐23.35
Low	22	859989	13.48	11.57‐15.39
HPV Assay Method				
PCR & hybridization	57	1167563	16.55	14.53‐18.56
Sequencing or real‐time PCR	12	337104	11.73	9.34‐14.11
HPV types				
High risk HPV	66	1426787	11.93	10.93‐12.92
Low risk HPV	46	1176995	2.78	2.35‐3.20
HPV infection types				
Single infection	35	1424052	11.00	9.32‐12.68
Multiple infection	48	1419715	3.44	2.69‐4.18
Dual infection	14	445316	2.30	2.01‐2.58
Triple infection	13	442280	0.51	0.42‐0.60
Fourfold infection	9	406238	0.11	0.07‐0.15
Fivefold infection	5	204499	0.04	0.01‐0.07
The top 10 subtypes				
HPV 16	68	1604203	3.52	3.18‐3.86
HPV52	65	1574238	2.20	1.93‐2.46
HPV58	65	1579921	2.10	1.88‐2.32
HPV18	60	1566134	1.20	1.05‐1.35
HPV33	54	1524449	1.02	0.89‐1.14
HPV31	52	1467424	0.84	0.74‐0.95
HPV51	50	1487714	0.72	0.64‐0.81
HPV 56	49	1415424	0.66	0.57‐0.74
HPV6	48	1500726	0.63	0.48‐0.78
HPV11	45	1506233	0.56	0.43‐0.69

**Table 3:** The pooled prevalence of Human papillomavirus (HPV) infection by age group

Age group	Number of databases	Number of Subjects	Prevalence (%)	95% CI
Group 1				
<25	4	3273	13.31	7.33‐19.28
25‐29	4	13619	9.97	6.89‐13.04
30‐34	4	22629	9.50	6.53‐12.47
35‐39	4	27780	10.38	7.66‐13.10
40‐44	4	30320	10.58	7.97‐13.19
45‐49	4	26892	10.24	8.19‐12.29
50‐54	4	32050	12.29	10.45‐14.13
55‐59	2	31302	14.93	7.96‐21.90
60‐65	2	10953	16.33	3.01‐29.65
66‐70	1	5302	10.37	9.55‐11.19
Group 2				
<25	3	876	19.21	10.62‐27.79
25‐34	3	6463	9.95	3.46‐16.43
35‐44	3	8782	12.54	7.26‐17.82
45‐54	2	1900	12.03	3.14‐20.92
55‐64	2	270	9.84	2.51‐17.18
>65	1	30	13.33	1.17‐25.49
Group 3				
<20	2	1732	33.37	17.67‐49.07
21‐30	5	31403	14.65	8.40‐20.89
31‐40	5	48829	13.94	8.03‐19.85
41‐50	5	52141	13.86	7.58‐20.14
51‐60	4	21496	18.89	10.74‐27.03
>60	2	2342	26.03	24.26‐27.81
