# Supplementary material for: The prevalence, trends, and geographical distribution of human papillomavirus infection in China: The pooled analysis of 1.7 million women
Source: Cancer Med. 2019 Jul 27;8(11):5373–85. doi: 10.1002/cam4.2017 (PMC6718589; doi:10.1002/cam4.2017)
Supplement: Supplementary file 1 [file CAM4-8-5373-s001.docx]

**Supplementary 1: The Modified Quality Assessment tool**

1. **was the study’s target population a close representation of the national population in relation to relevant variables?**

 Yes: The study’s target population was a close representation of the national population. (1 point)

 No: The study’s target population was clearly NOT representative of the national population. (0 point)

1. **was the sampling frame a true or close representation of the target population?**

 Yes: The sampling frame was a true or close representation of the target population. (1 point)

 No: The sampling frame was NOT a true or close representation of the target population. (0 point)

1. **was some form of random selection used to select the sample, OR, was a census undertaken?**

 Yes: A census was undertaken, OR, some form of random selection was used to select the sample (e.g. simple random sampling, stratified random sampling, cluster sampling, systematic sampling). (1 point)

 No: A census was NOT undertaken, AND some form of random selection was NOT used to select the sample. (0 point)

1. **was the likelihood of non-response bias minimal?**

 Yes: The response rate for the study was >/=75%, OR, an analysis was performed that showed no significant difference in relevant demographic characteristics between responders and nonresponders. (1 point)

 No: The response rate was <75%, and if any analysis comparing responders and non-responders was done, it showed a significant difference in relevant demographic characteristics between responders and non-responders. (0 point)

1. **were data collected directly from the subjects?**

 Yes: All data were collected directly from the subjects. (1 point)

 No: In some instances, data were collected from a proxy. (0 point)

1. **was an acceptable case definition used in the study?**

 Yes: An acceptable case definition was used. (1 point)

 No: An acceptable case definition was NOT used. (0 point)

1. **was the study instrument that measured the parameter of interest (e.g. prevalence of low back pain) shown to have reliability and validity (if necessary)?**

 Yes: The study instrument had been shown to have reliability and validity (if this was necessary), e.g. test-retest, piloting, validation in a previous study, etc. (1 point)

 No: The study instrument had NOT been shown to have reliability or validity (if this was necessary). (0 point)

1. **was the same mode of data collection used for all subjects?**

 Yes: The same mode of data collection was used for all subjects. (1 point)

 No: The same mode of data collection was NOT used for all subjects. (0 point)

1. **was the length of the shortest prevalence period for the parameter of interest appropriate?**

 Yes: The shortest prevalence period for the parameter of interest was appropriate (e.g. point prevalence, one-week prevalence, one-year prevalence). (1 point)

 No: The shortest prevalence period for the parameter of interest was not appropriate (e.g. lifetime prevalence) (0 point)

1. **were the numerator(s) and denominator(s) for the parameter of interest appropriate?**

 Yes: The paper presented appropriate numerator(s) AND denominator(s) for the parameter of interest (e.g. the prevalence of low back pain). (1 point)

 No: The paper did present numerator(s) AND denominator(s) for the parameter of interest but one or more of these were inappropriate. (0 point)

Total maximum 10 points
