# Supplementary material for: The prevalence, trends, and geographical distribution of human papillomavirus infection in China: The pooled analysis of 1.7 million women
Source: Cancer Med. 2019 Jul 27;8(11):5373–85. doi: 10.1002/cam4.2017 (PMC6718589; doi:10.1002/cam4.2017)
Supplement: Supplementary file 2 [file CAM4-8-5373-s002.docx]

**Supplementary 2. The References of the included studies**

1. Jin LM, Zhang XH, Xiao LP, et al. Genotyping analysis of human papillomavirus infection in cervix uteri among 116 441 census women. *Journal of Bengbu Medical College.* 2018;43: 462-7.

2. Zhang C, Zhang C, Huang J, et al. Prevalence and genotype distribution of human papillomavirus among females in the suburb of Shanghai, China. *Journal of medical virology.* 2018;90: 157-64.

3. Wang X, Ji Y, Li J, et al. Prevalence of human papillomavirus infection in women in the Autonomous Region of Inner Mongolia: A population-based study of a Chinese ethnic minority. *Journal of medical virology.* 2018;90: 148-56.

4. Dong L, Hu SY, Zhang Q, et al. [Changes in genotype prevalence of human papillomavirus over 10-year follow-up of a cervical cancer screening cohort]. *Zhonghua liu xing bing xue za zhi = Zhonghua liuxingbingxue zazhi.* 2017;38: 20-5.

5. Zhou XH, Shi YF, Wang LJ, et al. Distribution Characteristics of Human Papillomavirus Infection: A Study Based on Data from Physical Examination. *Asian Pacific journal of cancer prevention : APJCP.* 2017;18: 1875-9.

6. Xu HH, Lin A, Chen YH, et al. Prevalence characteristics of cervical human papillomavirus (HPV) genotypes in the Taizhou area, China: a cross-sectional study of 37 967 women from the general population. *BMJ open.* 2017;7: e014135.

7. Zhong TY, Zhou JC, Hu R, et al. Prevalence of human papillomavirus infection among 71,435 women in Jiangxi Province, China. *Journal of infection and public health.* 2017;10: 783-8.

8. Wu X, Zhao J, Cui XL, et al. [Prevalence of type-specific human papillomavirus infection among 18-45 year-old women from the general population in Liuzhou, Guangxi Zhuang Autonomous Region: a cross-sectional study]. *Zhonghua liu xing bing xue za zhi = Zhonghua liuxingbingxue zazhi.* 2017;38: 467-71.

9. Abulizi G, Li H, Mijiti P, et al. Risk factors for human papillomavirus infection prevalent among Uyghur women from Xinjiang, China. *Oncotarget.* 2017;8: 97955-64.

10. Zhang JY, Wang WJ, Yang SZ, et al. Investigation of cervical papillomavirus infection in 58650 women in Chengdu. *Shandong Medical Journal.* 2017: 65-7.

11. Qi ZY, Sun P, Xu JH, et al. Study on the status and intervention measures of HPV infection in Chengde. *Hebei Medical Journal.* 2017;39: 769-72.

12. Shen XP, Min QH, Wu D, et al. Screening of papillomavirus in 20 thousand rural women in Ningxia. *Ningxia Medical Journal.* 2017;39: 363-4.

13. Zhang XH, Li FL, Li DH, et al. Epidemiological status of cervical HPV infection in women in Shaanxi province. *Chinese Journal of Woman and Child Health Research.* 2017: 1589-92.

14. Yang DM, Xia R, Lei L. Human papillomavirus infection in women with smooth cervix in Changsha institutions. *Chinese Journal of Virology.* 2017: 848-53.

15. Jin DC, Guo YH. Epidemiological Features and Analysis of Human Papilloma Virus Infection among Female Residents of Zhengzhou. *J Med Res.* 2017: 88-92.

16. Baloch Z, Li Y, Yuan T, et al. Epidemiologic characterization of human papillomavirus (HPV) infection in various regions of Yunnan Province of China. *BMC infectious diseases.* 2016;16: 228.

17. Gu Y, Yi M, Xu Y, et al. Genotype distribution characteristics of high-risk human papillomaviruses in women from Shanghai, China. *Epidemiology and infection.* 2016;144: 1482-9.

18. Liu F, Deng Q, Zhang C, et al. Human papillomavirus DNA positivity and seropositivity in rural Chinese men and women: a population-based cross-sectional study. *Scientific reports.* 2016;6: 26343.

19. Wei F, Yin K, Wu X, et al. Human papillomavirus prevalence and associated factors in women and men in south China: a population-based study. *Emerging microbes & infections.* 2016;5: e119.

20. Zeng XX, Yan LX, Huang XX, et al. Prevalence and genotype distribution of human papillomavirus among Hakka women in China. *Annals of translational medicine.* 2016;4: 276.

21. Niyazi M, Husaiyin S, Han L, et al. Prevalence of and risk factors for high-risk human papillomavirus infection: a population-based study from Hetian, Xinjiang, China. *Bosnian journal of basic medical sciences.* 2016;16: 46-51.

22. Zhang Y, Wang Y, Liu L, et al. Prevalence of human papillomavirus infection and genotyping for population-based cervical screening in developed regions in China. *Oncotarget.* 2016;7: 62411-24.

23. Que M, Sun QL, Cheng GC, et al. Prevolence of human papillomavirus infection and cervical lesions among rural women in five counties of Anhui province. *Chin J Public Health.* 2016;32: 493-7.

24. Ji HY. The Analysis of human papillomavirus Genotypein about Han,Tibetan,Hui women living in Qinghai area: Qinghai University, 2016.

25. Long X, Zhou DP, Yang J, et al. Analysis of the Distribution Characteristics of Human Papilloma Virus Infection Among 29580 Females in Chongqing Area. *Journal of Practical Obstetrics and Gynecology.* 2016;32: 464-7.

26. Wang JX. The Analysis of human papillomavirus infection in cervix of women in Wuxi City in 2013. *Maternal and Child Health Care of China.* 2015: 4363-6.

27. Mijit F, Ablimit T, Abduxkur G, et al. Distribution of human papillomavirus (HPV) genotypes detected by routine pap smear in Uyghur-Muslim women from Karasay Township Hotan (Xinjiang, China). *Journal of medical virology.* 2015;87: 1960-5.

28. Zhao Y, Zhao F, Hu S, et al. [Multi-center cross-sectional study on type-specific human papillomavirus infection among Chinese women]. *Zhonghua liu xing bing xue za zhi = Zhonghua liuxingbingxue zazhi.* 2015;36: 1351-6.

29. Chen X, Wallin KL, Duan M, et al. Prevalence and genotype distribution of cervical human papillomavirus (HPV) among women in urban Tianjin, China. *Journal of medical virology.* 2015;87: 1966-72.

30. Hong H, He TF, Ni HX, et al. Prevalence and genotype distribution of HPV infection among women in Ningbo, China. *International journal of gynaecology and obstetrics: the official organ of the International Federation of Gynaecology and Obstetrics.* 2015;131: 96-9.

31. Yang L, He Z, Huang XY, et al. Prevalence of human papillomavirus and the correlation of HPV infection with cervical disease in Weihai, China. *European journal of gynaecological oncology.* 2015;36: 73-7.

32. Zhao Q, Chen ZH, Zhu XL, et al. Analysis of the screening result of cervical lesions in 24817 women undergone health examination in Hunan province. *Basic & Clinical Medicine.* 2015;35: 435-8.

33. Li XL, Zhang XG, Li HY. Analysis of human papillomavirus infection characteristics among women of Yichang area. *CHIN J CANCER PREV TREAT.* 2015: 1261-5.

34. Chen MH, Yin YX, Zhang YJ, et al. A retrospective analysis of the HPV genotyping of 11231 women's cervix. *Maternal and Child Health Care of China.* 2014: 5570-3.

35. Jing L, Zhong X, Zhong Z, et al. Prevalence of human papillomavirus infection in Guangdong Province, China: a population-based survey of 78,355 women. *Sexually transmitted diseases.* 2014;41: 732-8.

36. Zhu MH, Lin CJ, Ye SY, et al. The epidemiological investigation on women of childbearing age with HPV infection and cervical lesions among floating population in Huizhou of Guangdong Province. *Chinese Journal of Family Plannning & Gynecotokology.* 2014: 25-8.

37. Pei YY, Li GC, Ran J, et al. Analysis of human papillomavirus infection in Longgang District of Shenzhen City. *Maternal and Child Health Care of China.* 2014: 3380-1.

38. Yang L, Li N, Guo LW, et al. [Prevalence of human papilloma virus and analysis of its risk factors in Daqing city, Heilongjiang province in 2010]. *Zhonghua yu fang yi xue za zhi [Chinese journal of preventive medicine].* 2013;47: 118-23.

39. Wu EQ, Liu B, Cui JF, et al. Prevalence of type-specific human papillomavirus and pap results in Chinese women: a multi-center, population-based cross-sectional study. *Cancer causes & control : CCC.* 2013;24: 795-803.

40. Zhang R, Shi TY, Ren Y, et al. Risk factors for human papillomavirus infection in Shanghai suburbs: a population-based study with 10,000 women. *Journal of clinical virology : the official publication of the Pan American Society for Clinical Virology.* 2013;58: 144-8.

41. Wu XQ, Tan J, Li YY, et al. Epidemiological survey on HPV infection and cervical lesions of floating women with childbearing age in Baiyun District of Guangzhou City. *Chinese Journal of Family Plannning & Gynecotokology.* 2013: 22-5.

42. Weng ZC, Zhu ZY, Jiang YQ, et al. Molecular epidemiology of human papillomavirus infection in Haikou. *Natural Science Journal of Hainan University.* 2013: 41-4.

43. Wu D, Cheng B, Liu S, et al. Study on human papillomavirus genotypes among cervical cells of female patients. *Chin Med Herald.* 2013: 123-5.

44. Wang S, Wei H, Wang N, et al. The prevalence and role of human papillomavirus genotypes in primary cervical screening in the northeast of China. *BMC cancer.* 2012;12: 160.

45. Wang Y, Huang Y, Chen Y. The epidemiologic study on human papillomavirus in Fujian province of China. *Lab Med Clin.* 2012: 3099-101.

46. Zhang J, Ruan CL. North region of Shaanxi asymptomatic femaIe population HPV infection situation and main type anaIysis. *Chinese JoumaI of Aesmetic Medicine.* 2012: 437-8.

47. Chen JC, Yang XS, Xu SW, et al. Epidemiological investigation on human papillomavirus nucleic acid genotyping in townswomen of Shantou city. *Lab Med Clin.* 2012: 1681-3.

48. Wang YY, Peng J, Zhou HB, et al. Molecular Epidemiology Analysis of Cervical Human Papilloma Virus Infection among Women in Shenzhen City. *Chin J Prev Contr Chron Dis.* 2012: 293-5.

49. Zhao R, Zhang WY, Zhang SW, et al. Study on subtype of human papillomavirus infection among aged 25—54 reproductive women in Beijing from 2006 to 2008. *Chin J Obstet Gynecol.* 2011: 184-7.

50. Liu SS, Chan KY, Leung RC, et al. Prevalence and risk factors of Human Papillomavirus (HPV) infection in southern Chinese women - a population-based study. *PloS one.* 2011;6: e19244.

51. Ablimit T, Turgan M, Abliz G, et al. [Study on the distribution of HPV subtypes in Uighur people living in the Karsay township, Moyu county, Xinjiang]. *Chin J Epidemiol.* 2011;32: 477-80.

52. Liu JH, Mo Y, Bai H, et al. Epidemiological Investigation of the Prevalence of High Risk Human Papillomavirus and Its Correlation with Cervical IntraepitheliaI Neoplasia in 4874 Women in Guangxi Province. *Journal of Practical Obstetrics and Gynecology.* 2011: 596-9.

53. Chen HC, You SL, Hsieh CY, et al. Prevalence of genotype-specific human papillomavirus infection and cervical neoplasia in Taiwan: a community-based survey of 10,602 women. *International journal of cancer.* 2011;128: 1192-203.

54. Yip YC, Ngai KL, Vong HT, et al. Prevalence and genotype distribution of cervical human papillomavirus infection in Macao. *Journal of medical virology.* 2010;82: 1724-9.

55. Ye J, Cheng X, Chen X, et al. Prevalence and risk profile of cervical Human papillomavirus infection in Zhejiang Province, southeast China: a population-based study. *Virology journal.* 2010;7: 66.

56. Wu D, Cai L, Huang M, et al. Prevalence of genital human papillomavirus infection and genotypes among women from Fujian province, PR China. *European journal of obstetrics, gynecology, and reproductive biology.* 2010;151: 86-90.

57. Jiang Y, Li Q, Li J, et al. Epidemiological Investigation on Human Papillomavirus Infection in women in Southern Fujian. *Modern Preventive Medicine.* 2010: 110-1+8.

58. Zou Y, Guo XZ, Chen J, et al. Typing and epidemiological analysis of human papillomavirus on female genital tract in Zhejiang province. *Chin J Epidemiol.* 2010: 353-4.

59. Jin Q, Shen K, Li H, et al. [Prevalence of human papillomavirus infection in women in Tibet Autonomous Region of China.]. *Zhonghua fu chan ke za zhi.* 2009;44: 898-902.

60. Li N, Dai M. Human papillomavirus infection in China：a multi-centric cross-sectional study. *Chin J Dis Control Prev.* 2008: 411-5.

61. Chao A, Hsu KH, Lai CH, et al. Cervical cancer screening program integrating Pap smear and HPV DNA testing: a population-based study. *International journal of cancer.* 2008;122: 2835-41.

62. Zhang X, Wang CY, Shi JF, et al. Study on the prevalence of human papillomavirus infection and distribution of types in Shenyang city. *Chin J Epidemiol.* 2007: 954-7.

63. Dai M, Bao YP, Li N, et al. Human papillomavirus infection in Shanxi Province, People's Republic of China: a population-based study. *British journal of cancer.* 2006;95: 96-101.

64. Li LK, Dai M, Clifford GM, et al. Human papillomavirus infection in Shenyang City, People's Republic of China: A population-based study. *British journal of cancer.* 2006;95: 1593-7.

65. Shi JF, Wu RF, Liu ZH, et al. Distribution of Human papillomavirus Types in Shenzhen women. *Acta Academiae Medicinae Sinicae.* 2006: 832-6.

66. Wu KH, Fu YG, Jiang H. Molecular epidemiological analysis of cervical HPV in women of childbearing age in Guangzhou. *Guangdong Medical Journal.* 2004: 405-6.

67. Chen F, Ceng ZP, Liu B, et al. Risk factors of human papillomavirus infection among women in Yangcheng County, Shanxi Province. *Journal of Practical Oncology.* 2004: 349-51.

68. Shen YH, Chen F, Huang MN, et al. Population-based study of human papillomavirus infection in high-risk area for cervical cancer in Shanxi Province, China. *Acta Academiae Medicinae Sinicae.* 2003: 381-5.
